# A Case of Lupus

**Published:** 1890-02

**Authors:** Langdon Frothingham


### A CASE OF LUPUS, OR CUTANEOUS TUBERCULOSIS,

By Langdon Frothingham, M.D.V.

The scientific men of to-day accept as the definition of tuberculosis any pathological change due to the presence of the bacillus of tuberculosis. In man, when this process is situated within the skin, the term lupus is applied, or, more correctly speaking, cutaneous tuberculosis.

So far as I know, this disease has not been described as affecting domestic animals. But there seems to be no reason why it should not attack any of them that are subject to tuberculosis. That the disease is rare among animals, as rare as it is in man, there is little doubt, but when it does exist its presence is doubly interesting to the veterinarian. On the one hand as another manifestation of a most dread disease, namely tuberculosis, and on the other that this form probably communicates tuberculosis to healthy animals more easily than any other. But of this I shall speak later. Of course, lupus may not necessarily communicate lupus, but some form of tuberculosis will be the probable result if the diseased animal be kept in the same barn and pasture as the healthy.

I have had the good fortune to see one case to which I do not hesitate to apply the term lupus, and which I will endeavor to describe :—

CASE.—A Grade Guernsey cow, supposed to be suffering slightly from tuberculosis. The respiration was thickened, thought to be due to an enlarged gland in the region of the larynx. There was but a slight cough. The animal gave a good quantity of milk, and her general health was good. There were several swellings or nodules on different parts of the body. Two were between the udder and vulva, one on the under surface of the tail, and several about the neck. They were about the size of a small horse-chestnut. When felt of, they appeared to be firm, hard nodules not unlike fibromata, and seemed to be within the skin.

Thinking that this was some tuberculous process, perhaps enlarged lymphatic glands, I was anxious to excise one of these nodules, cut sections and examine them for the bacilli of tuberculosis. This, however, I was not permitted to do. Left to itself the disease ran its own course. These hard, firm nodules gradually became soft and finally suppurated, discharging a thick, cheesy matter which collected on the hairs and formed a thick, yellow crust. Matters were now reversed, the nodule in the skin became an excrescence, with a well-marked apex and base, a more or less firm crust under which was a mass of soft, cheesy matter. This whole excrescence might have been easily detached, and might thus have hastened the healing process. But left to itself the crust and cheesy matter gradually disappeared, leaving a small cicatrix.

To make a positive diagnosis of lupus it was of course necessary to demonstrate the presence of the bacilli of tuberculosis. An incision was therefore made through the crust of one of the excrescences, and taking some fresh cheesy material from the centre, covered glass preparations were made in the usual manner. These were stained by the Ehrlich Koch's twenty-four hour method and mounted. Upon examination under the microscope bacilli of tuberculosis were shown to be present. This proves without doubt that the above case was one of lupus.

Before closing, let me say a few words in regard to the risk that people run by allowing animals to exist that are suffering from tuberculosis or lupus.

*First*—TUBERCULOSIS.—Every one to-day knows the contagious nature of this disease. No matter where the tubercle may be situated, sooner or later it becomes a source of danger among us. This may sound like a very sweeping and irrational statement to some ; therefore, a few facts may be necessary to corroborate it. The disease may be communicated from animal to animal, from the diseased to the healthy. It may spread to different parts of the same animal. One may easily prove this by observation and by inoculation experiments. Since almost every portion of a steer or cow may be used for human food, if the glandular organs, liver, spleen, &c., are the seats of the tubercle, there is danger. The milk of tuberculous cows is dangerous, for the bacillus of tuberculosis may be demonstrated in such milk, and if this milk or cream be introduced under the skin of rabbits and guinea pigs a large percentage of the results will prove positive, that is to say, tuberculosis will be the result. This then, very briefly, is, I think, enough to warrant the above statement.

*Second*—LUPUS.—If it be borne in mind that during what may be called the ulcerative stage, masses of tubercle bacilli are exposed on the surface of the body, one does not need a very vivid imagination to see at a glance the various ways that tuberculosis may spread from this as a centre. A few may be inserted. Other animals in the herd may lick the sores. The man tending the animal may handle the tubercle after it has suppurated, and if he does not wash his hands thoroughly, or worse still, has a cut on his hand and gets some of the cheesy material into it, is not this dangerous? Again, suppose the cheesy degenerated mass is detached and thrown, naturally enough, into the pig pen. Is it not more than likely that the pigs or chickens may devour it, and in time become tuberculous? Then they are killed for human food. Thus the danger becomes as imminent to human beings as to animals.

Of course this is only supposition, but what *may* happen ought to be prevented; therefore, get rid of your original source of contagion. May the time not be far distant when the State shall give us instructions to kill every animal, perhaps more especially cattle, suffering from tuberculosis.

